# The Vulgar Person

**Published:** 1919-05-24

**Authors:** 


### HUMAN TEMPERAMENTS.

### THE VULGAR PERSON.

"Are you looking for a sharp knife, sir?" said the waiter to a guest who had left the table and was rummaging among the knives on the sideboard. "Allow me to sharpen one for you."

"No, you fool," replied the guest, "I am looking for a blunt one. Can't you see that I have just cut my mouth?"

A vulgar man to put his knife into his mouth! Certainly. It is vulgar because it is considered vulgar, because there is a conventional and arbitrary rule against it, because it is one of those things against which polite society sets a face of flint; but the inherent vulgarity of the guest was displayed not nearly as much in putting his knife into his mouth as in calling the waiter a fool. The first is conventionally vulgar, and on a change of fashion may become the height of good breeding; the second is inherently vulgar, and no change of fashion could deprive it of its vulgarity.

There are two kinds of vulgarity, therefore—conventional vulgarity and inherent vulgarity, or vulgarity of manner and vulgarity of soul; and of these the first is trivial and curable, the second is serious and incurable.

Vulgarity of manner consists in the breach of conventional regulations—some of them very absurd—that have been enacted as inflexible rules, and are, in fact, mere fashions, as irrational as most other fashions, and subject to the fluctuations and changes that affect all fashions. If they were all reversed to-morrow no one would be any the worse, except that those who are now in the height of the fashion would be considered vulgar, but this would be balanced by the advantage of those who are now considered vulgar and would then become fashionable.

Vulgarity of manner attaches only to trivial acts, such as the pronunciation of words and peculiarities of dress, and they are violations of laws that rest upon no principle, but are mere whimsies, conformance with which serves to show familiarity with a certain class of society, and shows no more.

To call a clerk a "clurk" is gross vulgarity, for the word retains its old pronunciation. To call a girl a "gurl" while not actually vulgar, does show a lack of acquaintance with the usages of the arbiters of polite pronunciation. On the other hand, no suspicion of vulgarity attaches to calling the clergy the "clurgy," although the world is so very closely allied to "clerk." The aforementioned arbiters have decided that the old pronunciation of "clerk" must be retained, and that the old pronunciation of "clergy" must be altered; but it is manifest that the decision does not depend upon reason. It is pure fashion. Very likely the vulgar have, in the case of "clurk," anticipated a change that the refined will make in time.

To omit the "h" at the beginning of a word is an offensive vulgarism; but to omit the "g" at the end of a word is the height of refinement. Manifestly there is no reason in this: it is pure convention.

In these matters of what may be called modal vulgarity it is often difficult to distinguish between vulgarity, archaism, provincialism, pedantry, and eccentricity. To call a clerk a "clurk" is vulgar; to call the clergy the "clargy" is archaic. To say "I 'ave done it" is vulgar. To say "Ah ov doon it" is slightly vulgar, but mainly provincial; and to say "I hev done it" is provincial without any vulgarity. Not to sound the final "e" in "Lalage" is vulgar. To sound it in "diatribe" is pedantic.

Modal vulgarity, as I have called it, displays itself in the utterance of words more, perhaps, than in anything else; but it is shown in many other things, all trivial and without inherent importance. There is no vulgarity in wearing round your hat a ribbon not tied by yourself; but it is vulgar to wear round your neck a cravat you have not yourself tied, or that has not been tied by your valet. However comfortable it may be, you must not wear a straw hat with a frock-coat. It is vulgar. So, too, it is vulgar to give your card to the footman when you enter a private house, but it is not vulgar to give your card to the messenger when you enter an office.

The odd thing is that, though all these vulgarities of manner are trivialities, and do not detract in the slightest from the worth of the person who displays them, yet they are usually considered far more important than the vulgarities of the soul, which are infallible indications of a debased nature.

One of these major vulgarities is to attach more importance to vulgarities of manner than to vulgarity of mind. While we are in Rome, we should do as the Romans do, and if our associates are so foolish as to think better of us if we call a table-napkin by this, its proper name, than if we call it a serviette, there is no reason why we should not bow ourselves in the House of Rimmon, and take the credit so easily gained; but what are we to think of people who attach importance to trifles like this, and see nothing vulgar in behaviour that hurts the feelings of other people, and betrays their own depravity of soul? True vulgarity consists in giving ourselves airs, in affectation, and in hurting, without provocation, the feelings of other people.

True vulgarity is inconsistent with naturalness of demeanour: true good manners are inconsistent with self-consciousness. It is the self-conscious person, the person who is perpetually speculating how his demeanour appears to other people, the person who is consciously acting for the purpose of impressing other people, who exhibits vulgarity of mind. The person who gives himself airs when he finds himself in a position superior to what he is accustomed to; the person who looks round for applause; the person who is on tenterhooks to

**Human Temperament—(*continued*).**

shove himself forward; the person who does not appreciate that he is not wanted; the person who takes to himself more than his rightful share, whether of meat from the dish or of attention from the company; he is the true vulgarian. Beyond this there are deeper depths of vulgarity. One of the lowest forms of vulgarity is to make cattish remarks about other people, present or absent; and the lowest depth of all is reached when a position of authority is used to inflict humiliation and mortification upon a subordinate. Modal vulgarity can be cured by care, attention, and education: vulgarity of soul is inherent and incurable. The modal vulgarian may know that he is vulgar, and strive successfully to cure himself. The vulgar in soul are self-satisfied, and never realise their baseness.

